# Altitude and COVID‐19: Friend or foe? A narrative review

**DOI:** 10.14814/phy2.14615

**Published:** 2020-12-19

**Authors:** Grégoire P. Millet, Tadej Debevec, Franck Brocherie, Martin Burtscher, Johannes Burtscher

**Affiliations:** ^1^ Institute of sport Sciences University of Lausanne Lausanne Switzerland; ^2^ Faculty of Sport University of Ljubljana Ljubljana Slovenia; ^3^ French Institute of Sport (INSEP) Paris France; ^4^ University of Innsbruck Innsbruck Austria

**Keywords:** coronavirus, hypoxemia, hypoxia, immunity, mitochondria, pandemic

## Abstract

Recent reports suggest that high‐altitude residence may be beneficial in the novel coronavirus disease (COVID‐19) implicating that traveling to high places or using hypoxic conditioning thus could be favorable as well. Physiological high‐altitude characteristics and symptoms of altitude illnesses furthermore seem similar to several pathologies associated with COVID‐19. As a consequence, high altitude and hypoxia research and related clinical practices are discussed for potential applications in COVID‐19 prevention and treatment. We summarize the currently available evidence on the relationship between altitude/hypoxia conditions and COVID‐19 epidemiology and pathophysiology. The potential for treatment strategies used for altitude illnesses is evaluated. Symptomatic overlaps in the pathophysiology of COVID‐19 induced ARDS and high altitude illnesses (i.e., hypoxemia, dyspnea…) have been reported but are also common to other pathologies (i.e., heart failure, pulmonary embolism, COPD…). Most treatments of altitude illnesses have limited value and may even be detrimental in COVID‐19. Some may be efficient, potentially the corticosteroid dexamethasone. Physiological adaptations to altitude/hypoxia can exert diverse effects, depending on the constitution of the target individual and the hypoxic dose. In healthy individuals, they may optimize oxygen supply and increase mitochondrial, antioxidant, and immune system function. It is highly debated if these physiological responses to hypoxia overlap in many instances with SARS‐CoV‐2 infection and may exert preventive effects under very specific conditions. The temporal overlap of SARS‐CoV‐2 infection and exposure to altitude/hypoxia may be detrimental. No evidence‐based knowledge is presently available on whether and how altitude/hypoxia may prevent, treat or aggravate COVID‐19. The reported lower incidence and mortality of COVID‐19 in high‐altitude places remain to be confirmed. High‐altitude illnesses and COVID‐19 pathologies exhibit clear pathophysiological differences. While potentially effective as a prophylactic measure, altitude/hypoxia is likely associated with elevated risks for patients with COVID‐19. Altogether, the different points discussed in this review are of possibly some relevance for individuals who aim to reach high‐altitude areas. However, due to the ever‐changing state of understanding of COVID‐19, all points discussed in this review may be out of date at the time of its publication.

## Graphical abstract

We reviewed the differences and similarities in the pathological responses, symptoms, mechanisms, treatments associated to altitude and to COVID‐19.

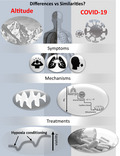

## 1 INTRODUCTION

Several similarities have been reported between respiratory responses/symptoms observed at high altitude and those associated with the novel coronavirus disease (COVID‐19), caused by the severe acute respiratory syndrome Coronavirus 2 (SARS‐CoV‐2): decrease in arterial oxygen partial pressure (PaO_2_) (i.e., hypoxemia), hyperventilation‐induced reduction of carbon dioxide partial pressure (i.e., hypocapnia) and the development of acute respiratory distress syndrome (ARDS) in severe cases (Geier & Geier, 2020; Solaimanzadeh, 2020a). However, despite overlaps in the pathophysiology of COVID‐19 induced ARDS and high altitude illnesses, these symptoms are also common in other pathologies (i.e., heart failure, pulmonary embolism, COPD…).

Understandably these parallels heightened interest and provoked speculations on the possible relationships between altitude and COVID‐19. However, to date, the effects, risks, and/or benefits of altitude/hypoxic exposure to prevent SARS‐CoV‐2 infection or to treat infected individuals remain unknown and untested. While some similar consequences unequivocally exist between altitude and COVID‐19‐induced hypoxia such as modified breathing patterns and systemic hypoxemia, disagreements exist on the five following points:

1. Do high‐altitude illnesses and SARS‐CoV‐2 induced pathologies share similar mechanisms?
2. Are the medications used for high‐altitude illnesses effective for treating COVID‐19?
3. Is COVID‐19 prevalence lower in higher altitude regions?
4. Might exposure to altitude/hypoxia provide a potential treatment for patients with COVID‐19? Are there any risks and what are the potential therapeutic benefits in mildly ill hypoxemic patients?
5. May hypoxic (pre‐)conditioning be beneficial for patients or individuals during the COVID‐19 pandemic?

As a group of physiologists investigating the ergogenic and therapeutic use of inspired hypoxia in obese (Fernandez Menendez et al., 2018), preterm born (Martin et al., 2020), COPD patients (Burtscher et al., 2010), elderly individuals (Millet et al., 2016b), and athletes (Millet et al., 2019), we aim to better understand the influences of the altitude‐induced (patho)physiological responses at the respiratory, arterial and mitochondrial level in relation to the COVID‐19 disease. We, therefore, reviewed the currently available literature in order to answer an apparently simple question: Are altitude and COVID‐19 friends or foes? All articles published in PubMed with the search criteria “COVID; altitude; SARS‐CoV‐2” at the date of 1st June 2020 have been reviewed. This question remains unanswered and the different points discussed in this review may support decision making for all individuals who aim to reach high‐altitude areas. Altogether, the different points discussed in this review are of possibly some relevance for individuals who aim to reach high‐altitude areas.

## 2 DO HIGH‐ALTITUDE ILLNESSES AND SARS‐COV‐2 INDUCED PATHOLOGIES SHARE THE SAME MECHANISMS?

There was an ongoing debate regarding similarities (Solaimanzadeh, 2020a, 2020b) or differences (Archer et al., 2020; Brugger et al., 2020; Luks et al., 2020; Luks & Swenson, 2020; Soliz et al., 2020) between COVID‐19 induced acute respiratory distress syndrome (ARDS) and high‐altitude pulmonary edema (HAPE) or acute mountain sickness (AMS).

Regarding AMS, aging has been proposed as a protective factor: individuals aged 50 years old and more are less likely to suffer from AMS at high‐altitude (Richalet et al., 2012). However, to our knowledge, no study has looked at the effect of aging on AMS using participants similar to the average age of death by COVID‐19 (>65 years old).

Solaimanzadeh (Solaimanzadeh, 2020a) first proposed a parallel between COVID‐19 and HAPE based on similarities in clinical symptoms: decreased ratio of PaO_2_ to fractional inspired oxygen (PaO_2_:FiO_2_ ratio), hypocapnia, and tachypnea. One of the most striking common features is profound hypoxemia in patients who display little breathlessness (i.e., called “happy hypoxemia” (Archer et al., 2020) or “hypoxemia without respiratory distress” (Gattinoni et al., 2020; Xie et al., 2020)). Similarities of clinical features, chest imaging, and bronchoalveolar lavage findings in later stages have also recently been emphasized (Luks et al., 2020; Luks & Swenson, 2020).

However, there is now a consensus that fundamentally different pathophysiological mechanisms underlie the COVID‐19 induced ARDS and HAPE and that both require different therapeutic approaches (Archer et al., 2020; Brugger et al., 2020; Luks et al., 2020; Luks & Swenson, 2020; Strapazzon et al., 2020).

HAPE is non‐cardiogenic pulmonary edema caused by exaggerated uneven hypoxic pulmonary vasoconstriction (HPV) and abnormally high mean pulmonary artery (PA) pressure leading to a non‐inflammatory, alveolar‐capillary leak and edema formation (Bartsch & Swenson, 2013; Maggiorini et al., 2001).

In contrast to HAPE, only modestly elevated PA pressure in ARDS constitutes a consequence rather than a cause (Luks & Swenson, 2020). The main characteristic in COVID‐19 induced ARDS is a profound cytokine‐mediated inflammatory response which, among others, can severely affect pulmonary gas exchange and capillary integrity (Luks et al., 2020). The coronavirus has been suggested to infect the respiratory epithelium by its interaction with membrane‐bound angiotensin‐converting enzyme 2 (ACE2) (Li et al., 2003). Moreover, ACE2 downregulation by SARS‐CoV‐2 spike protein and related deleterious effects of elevated angiotensin II and decreased angiotensin 1–7 have been proposed as an explanation of the cytokine storm and the multi‐organs dysfunction seen in COVID‐19 patients (Banu et al., 2020; Hoiland et al., 2020).

Even if arterial hypoxemia can have detrimental effects on the pulmonary vasculature, HAPE is mainly detrimental to the lungs, while COVID‐19 can affect all tissues (lungs, kidney, heart, central nervous system) expressing ACE‐2 receptors. ARDS related to SARS‐CoV‐2 infection exhibits distinct manifestations when compared to traditional ARDS; sometimes an absence of dyspnea (Archer et al., 2020; Gattinoni et al., 2020), relatively preserved lung compliance, large intrapulmonary shunt, pulmonary vessel abnormalities such as thrombosis, microangiopathy, and strongly increased angiogenesis (Ackermann et al., 2020). However, recent comparisons of ARDS and COVID‐19 reported also similarities; that is, the same range of reduced compliance, the same range of shunt, and thrombosis. Even if evidences are weak (Pun et al., 2020), a possibly reduced ACE‐2 expression as a form of altitude adaptation has been considered as being beneficial for COVID‐19 (Arias‐Reyes et al., 2020), although COVID‐19 risk appears to be unaffected by changed ACE‐2 expression in response to ACE inhibitors or angiotensin receptor blockers (Khera et al.,). Moderate changes in ACE‐2 levels thus do not seem to be associated with SARS‐CoV‐2 infection. Its complex modulatory role in the renin‐angiotensin system (RAS) (Rossi et al., 2020), the potential of soluble ACE‐2 to scavenge SARS‐CoV‐2 (Rossi et al., 2020), and a possible beneficial role of ACE‐2 in ARDS (Kuba et al., 2005), complicate the interpretation of reduced ACE‐2 levels at high altitude. Currently, however, there is no evidence for a reduced ACE‐2 expression in high‐altitude residents.

Comorbidity factors are also strikingly different between COVID‐19 and HAPE. Although age is an important risk factor and males are at higher risk for COVID‐19, it seems that aging may even be protective against AMS (Richalet & Lhuissier, 2015), since an increased ventilatory response to hypoxia results in attenuated desaturation, at least in males (Lhuissier et al., 2012).

Taken together, COVID‐19 ARDS and HAPE are clearly different in pathogenesis and pathophysiology, as summarized recently by Luks and Swenson (Luks & Swenson, 2020), Luks et al. (Luks et al., 2020), and Brugger et al. (Brugger et al., 2020). These authors conclude that *“the differences far outweigh the similarities”* between COVID‐19 and HAPE.

Finally, all these symptoms are also common in other pathologies (i.e., heart failure, pulmonary embolism, COPD…).

## 3 ARE THE MEDICATIONS AND METHODS USED FOR HIGH‐ALTITUDE ILLNESSES EFFECTIVE FOR TREATING COVID‐19?

Expectedly, the use of medications (e.g., corticosteroids, carbonic anhydrase inhibitors, calcium channel blockers (Solaimanzadeh, 2020b), erythropoietin (EPO) (Soliz et al., 2020)) or methods (e.g., supplemental oxygen, hyperbaric oxygen therapy (Geier & Geier, 2020)) for high‐altitude illnesses (HAPE or AMS) in COVID‐19 patients is also debated.

Supplemental oxygen supply is the therapy of choice in HAPE causing rapidly diminishing HPV and PA pressure followed by complete recovery within hours/days (Luks & Swenson, 2020). In COVID‐19 patients, supplemental oxygen therapy to target SpO_2_ in the range of 90%–95% is recommended in those suffering from severe respiratory distress (https://www.who.int/publications/i/item/clinical‐management‐of‐covid‐19).

The calcium channel blocker nifedipine or the phosphodiesterase‐5 inhibitors sildenafil and tadalafil are also able to reduce HPV and PA pressure and therefore are effective in the treatment of HAPE (Maggiorini, 2010). In ARDS, however, generalized pulmonary vasodilation provoked by these drugs may even aggravate hypoxemia due to increased perfusion of poorly ventilated lung regions (Luks et al., 2020; Luks & Swenson, 2020).

Despite its established role in the prevention of HAPE (Maggiorini et al., 2006) and the recent accumulation of data that it leads to a significant reduction in death for patients with severe symptoms, the use of corticosteroids in COVID‐19 pneumonia, and ARDS in general remains discussed in the scientific community (Russell et al., 2020). Very recently, however, a major breakthrough was promised by demonstrating large live‐saving effects for dexamethasone (a commonly used steroid) especially in critically ill COVID‐19 patients (https://www.nature.com/articles/d41586‐020‐01824‐5). The adrenal response to COVID‐19 infection is markedly increased, indicating a profound stress situation (Berton et al., 2020). Dexamethasone may suppress the immune system and thereby hamper the cytokine storm caused by an over‐active immune response in severely ill patients. COVID‐19 patients receiving dexamethasone must be closely monitored for potential adverse effects, including hyperglycemia, psychiatric symptoms, and secondary infections (i.e., bacterial, fungal, parasitic, and mycobacterial). Besides, prolonged treatment with dexamethasone may, except weight gain and fluid retention, be associated with the risk of reactivation of latent infections like herpesvirus, hepatitis B, or tuberculosis. Last but not least, Dexamethasone has myriad effects (e.g., reduction in vascular permeability, suppression of inflammatory pathways or sympatholysis) (Swenson, 2016) with unclear consequences on patients with COVID‐19. Therefore, attending physicians have to consider potential drug interactions (for COVID treatment guidelines see: https://www.covid19treatmentguidelines.nih.gov/immune‐based‐therapy/immunomodulators/corticosteroids/).

Exposure to hypoxia induces a hypoxic ventilatory response causing respiratory alkalosis. Carbonic anhydrase inhibitors (e.g., acetazolamide) offset the resulting braking effect on ventilation. Acetazolamide inhibits the reabsorption of bicarbonate, sodium, and chloride ions by the kidney and is the most common medication for preventing AMS (Swenson, 2016). While it has been recommended in COVID‐19 patients (Solaimanzadeh, 2020a), caution should be exercised in patients already dyspneic since acetazolamide can precipitate respiratory failure (Adamson & Swenson, 2017; Luks & Swenson, 2020). Moreover, acetazolamide is generally contraindicated in patients under mechanical ventilation; except in some circumstances,such as the correction of severe metabolic alkalosis (Adamson & Swenson, 2017).

The kidney produces EPO a circulating hormone that stimulates erythropoiesis (production of red blood cells) by binding and activating the EPO receptors on erythroid progenitor cells. Beyond this well‐known effect, there are also indirect (non‐erythropoietic) effects of EPO. For instance, it counteracts pulmonary vasoconstriction by increasing the endothelial capacity to produce the vasodilator nitric oxide (NO) (Beleslin‐Cokic et al., 2011).

EPO’s effects on increased erythropoiesis and heme synthesis have been proposed to potentially alleviate COVID‐19‐associated severe hypoxemic states, rendering EPO a promising potential adjuvant treatment (Soliz et al., 2020). This is very unlikely since COVID‐19 has been associated with the hyperferritinemic syndrome spectrum with the potential deleterious effect of the high level of ferritin and free iron (Perricone et al., 2020). Iron chelation and iron depletion therapy have been proposed as a novel therapeutic approach (Abobaker, 2020; Perricone et al., 2020) but this treatment is not supported by experimental or clinical results and remains speculative.

Hyperbaric oxygen therapy (HBOT) is known to be effective for HAPE and has been proposed as adjuvant treatment for COVID‐19 (Geier & Geier, 2020; Thibodeaux et al., 2020) since it increases the blood oxygen levels and may prevent or delay the need for mechanical ventilation. Anecdotal reports suggest that HBOT improves both, hypoxemia and tachypnea of the infected patients (Thibodeaux et al., 2020).

In summary, further research is warranted to explore the potential usefulness of the different high‐altitude illnesses treatments in COVID‐19 patients with respiratory conditions (Geier & Geier, 2020). Of particular clinical relevance may be the very new findings on dexamethasone effectiveness in severe COVID as this drug is also considered to be a panacea for the prevention of severe high‐altitude illnesses (Hackett & Roach, 2001; Maggiorini et al., 2006).

## 4 IS COVID‐19 PREVALENCE LOWER IN HIGH ALTITUDE REGIONS?

By comparing the epidemiological data between high‐ and low‐altitude areas (e.g., in the Tibetan region of China, in Bolivia and Ecuador), it was suggested that COVID‐19 prevalence may be lower in areas or regions at high altitude (Arias‐Reyes et al., 2020). This is in line with another report on a very small local COVID‐19 propagation in the Qinghai‐Tibetan plateau (Xi et al., 2020). Recently, it was also proposed that residential altitude would lower the infection rate but not the mortality (Segovia‐Juarez et al., 2020). A few potential mechanisms have been proposed: first, as previously mentioned (see point 1 and the lack of convincing evidence (Pun et al., 2020)), the altitude‐related decrease of the ACE2 expression, suggesting a protective effect against COVID‐19 ARDS (Arias‐Reyes et al., 2020); second, potentially protective effects of higher EPO levels in some altitude residents (Soliz et al., 2020); third, the higher level of ultraviolet radiation at an altitude that may hamper the survival of the virus (Arias‐Reyes et al., 2020); and fourth, the lower barometric pressure leading to a reduced air density that would lessen the viral dissemination between people (Arias‐Reyes et al., 2020). All these mechanisms currently lack sufficient evidence and remain speculative. As noted recently (Pun et al., 2020), “*any current observations regarding high altitude‐related differences in incidence, prevalence, and morbidity/mortality of COVID‐19 must be considered speculative*.” It is for example unknown how tourism impacts these factors. “*There is a multitude of other environmental, political, temporal, and healthcare system factors at play*.”

In general, the susceptibility of high‐altitude populations for COVID‐19 has been reported (Zeng et al., 2020). Furthermore, anecdotal reports suggest that some important clusters in Europe may arise from ski resorts in altitude (Correa‐Martinez et al., 2020). Others (including ourselves) urge caution regarding this purported benefit of high‐altitude residence and call for further comprehensive evaluation of potential altitude‐related effects also encompassing other (social, demographic) risk factors (Burtscher et al., 2020; Huamani et al., 2020; Pun et al., 2020). It is crucial to consider that different countries employ different diagnostic approaches. High‐altitude residence may also be deleterious (increased severity or mortality) for respiratory diseases (Burtscher, 2014; Luks & Swenson, 2007; Perez‐Padilla & Franco‐Marina, 2004). Moreover, intermittent hypoxic preconditioning can be used to improve the exercise capacity in COPD patients (Faulhaber et al., 2015) (see point 5). Respiratory disease linked to previous pandemics (e.g., H1N1) actually led to worse outcomes in altitude areas (Perez‐Padilla et al., 2013). Finally, altitude‐induced hypoxemia may be exacerbated by “happy hypoxemia” described in COVID‐19 and thereby directly worsen pneumonia or indirectly ARDS via increased respiratory muscle fatigue (Luks & Swenson, 2020).

## 5 MIGHT EXPOSURE TO ALTITUDE/HYPOXIA PROVIDE BENEFITS THROUGH HYPOXIA CONDITIONING EFFECTS?

Hypoxia may be both detrimental or protective to cells, organs, and organisms (Lee et al., 2019). Severity, duration, and frequency (collectively defining the overall hypoxic dose (Millet et al., 2016a)) are the major determinants of the subsequent physiological response to hypoxic exposure (Navarrete‐Opazo & Mitchell, 2014). While severe hypoxic episodes are involved in a number of pathologies, lower hypoxic doses administered in appropriate temporal patterns can be protective for a lot of physiological functions including the neuronal, cardiovascular, and immune systems. Hypoxic (pre)conditioning is characterized by repeated exposures to hypoxia at sub‐harmful levels aiming to induce adaptations that render cells and tissues less vulnerable to subsequent hypoxic insults (Noble, 1943). Crucial regulators of these adaptations are the hypoxia‐inducible factors (HIFs) (Almohanna & Wray, 2018) that can activate a large number of molecular effectors including EPO (Ruscher et al., 2002) and vascular endothelial growth factor (VEGF) (Sondell et al., 1999). The main protective molecular adaptations achieved by hypoxic conditioning are believed to comprise improved vascularization, antioxidant capacities, and bioenergetics.

Hypoxic conditioning, with the HIF‐pathways at its core, has the capacity to enhance endogenous cellular antioxidant capacities and mitochondrial efficiency, best investigated in heart (Jašová et al., 2017; Murphy & Steenbergen, 2007) and brain (Correia et al., 2010; Dirnagl & Meisel, 2008). In mouse models of brain injury, hypoxic preconditioning furthermore reduces inflammation and brain damage (Yin et al., 2007).

Induction of HIF‐pathways controls mitochondrial biogenesis, morphology, and respiration. Conversely, a number of mitochondrial metabolites—notably succinate—and reactive oxygen species (ROS) control the activation of HIFs (Fuhrmann & Brüne, 2017).

Acute exposure to high altitude (4300m) has also been shown to increase the plasma levels of lactate and succinate in humans (Tissot van Patot et al., 2009), suggesting the redirection of pyruvate to anaerobic energy production (glycolysis), inhibiting the tricarboxylic acid cycle (TCA)‐cycle and resulting in the accumulation of succinate (O'Brien et al., 2015). At the same time, glutathione was downregulated (Tissot van Patot et al., 2009), further strengthening the notion of ROS importantly mediating adaptations to hypoxia (Debevec et al., 2017).

**Figure 1:**
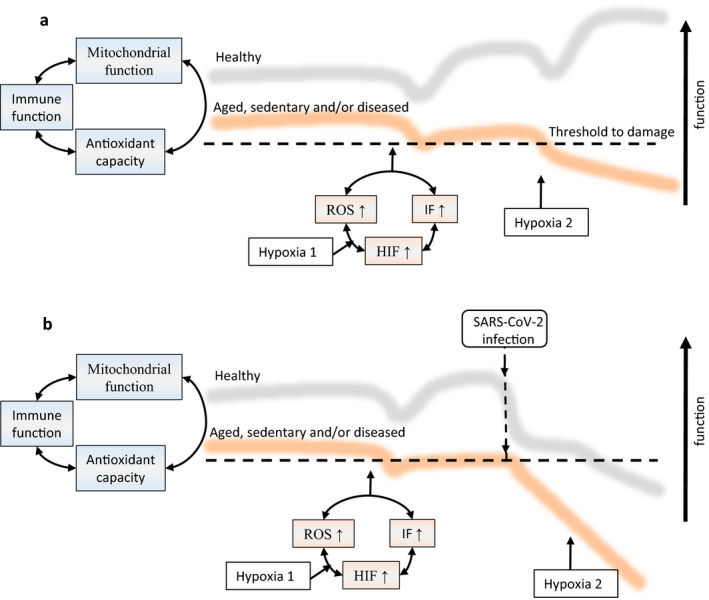
Benefits of hypoxic conditioning depend on the health of the organism. (a) A healthy organism (grey) cannot only buffer small hypoxic insults but will adapt by bolstering the inter‐related antioxidant capacities, immune and mitochondrial functions (hormesis) conferring increased tolerance to subsequent insults onto the organisms. Conversely, induction of intertwined reactive oxygen species (ROS) signaling and hypoxia‐inducible factors (HIF) and inflammation (IF) pathways by a single or repeated hypoxic stimulation may decrease the system's capacity below a threshold that induces long‐lasting damage. This may be the case, if the hypoxic insult is too severe or if the individual exhibits reduced mitochondrial, anti‐oxidant or anti‐inflammatory capacities. (b) Severe Acute Respiratory Syndrome Coronavirus 2 (SARS‐CoV‐2) infection may by itself induce sustained damage in vulnerable individuals or decrease the tolerance to subsequent insults

Taken together, hypoxic conditioning via induction of strongly inter‐related ROS signaling, HIF‐ and inflammation‐pathways (see next subsection) following mild hypoxic insults may strengthen mitochondria, antioxidant capacities, and immune function (see next subsection). If the related cellular buffer capacities to handle minor deficits in bioenergetics, antioxidant of inflammatory defenses of the system is reduced, for example by COVID‐19, even mild hypoxic insults may cause sustained damage (Figure 1).

In the following subsection, we will discuss the roles of mitochondria and inflammation in hypoxia and viral infection and the consequences of immunity and inflammation.

### 5.1 Viral infections, hypoxia, and mitochondria: molecular cross‐talk

Viral infections and immune responses leave their marks on cellular physiology and also on energy metabolism and mitochondria (Sander & Garaude, 2018). Mitochondrial damage has been demonstrated to follow infection by a number of RNA‐viruses (coronaviruses including SARS‐CoV‐2 are also RNA‐viruses). Localization of viral RNA in mitochondria and resulting mitochondrial dysfunction has been described for example for human immunodeficiency virus (HIV) (Somasundaran et al., 1994). The important role of functional mitochondria has also been clearly demonstrated in HIV infection as it is impaired in cells depleted of mitochondrial DNA (Lu et al., 2013). While experimental data on SARS‐CoV‐2 in that regard are still missing, its RNA is predicted to be imported into the host cells’ mitochondria as well (2020), likely damaging them and suggesting an important role of mitochondria in pathogenesis.

Mitochondria are also integral in the innate immune system. Especially the mitochondrial antiviral signaling (MAVS) complex plays a central role in linking cytoplasmic pathogen detection to transcriptional responses. This process importantly involves the induction of class I interferons and cytokines (Refolo et al., 2020). Healthy mitochondria and functional oxidative phosphorylation are essential for MAVS activation and the anti‐viral host defense (Koshiba et al., 2011; Yoshizumi et al., 2017). This is similar to influenza virus infection, where the virus reduces mitochondrial function to evade the cell's anti‐viral defenses by reducing mitochondrial function and thus MAVS activation (Varga et al., 2012).

Specialized immune cells also appear to require highly functional mitochondria in order to become activated (Sander & Garaude, 2018). Once activated, however, many pro‐inflammatory immune cells rely on glycolysis (Banoth & Cassel, 2018) that is accompanied by the activation of HIF‐1α (O'Neill et al., 2016).

Intriguingly, viral infection is commonly associated with higher reliance of cells on glycolysis for energy production. This effect is very apparent in dengue virus infection, which leads to an upregulation of glycolysis‐linked enzymes in the host cell favoring viral replication (Fontaine et al., 2015). Hepatitis C virus infection leads to reduced oxidative phosphorylation in the host cell, without loss of overall bioenergetics capacity. This is achieved by the viral induction of HIF‐1, increasing glycolysis (Ripoli et al., 2010). Moreover, increased HIF‐1 stabilization is associated with an enhanced anti‐viral defense through the upregulation of interferon and cytokine expression, and interferons and cytokines also induce HIF‐1 in an oxygen‐independent manner (Palazon et al., 2014). Hwang et al. (Hwang et al., 2006) demonstrated that the activation of HIF, indeed, protected carcinoma‐cells from vesicular stomatitis virus infection‐associated cytotoxicity. The interaction between HIF‐1 and inflammation suggests that while HIF‐1 is an inducer of anti‐viral defenses, it may at the same time create favorable environments for viral replication, including a switch to glycolysis and promotion of cell survival. In general, HIF‐1 and glycolytic metabolites are thought to promote inflammation (Ivashkiv, 2020). Hypoxia (that can also be induced by infection, inflammation or ischemic injury for example) and inflammation induce anaerobic and aerobic glycolysis, respectively, resulting in lactate accumulation and extracellular acidification (Ivashkiv, 2020). Lactate directly inhibits MAVS resulting in reduced class I interferon production (Zhang et al.,) and in addition, exerts immunosuppressive effects (Ivashkiv, 2020). Acidosis further inhibits the innate immune responses (Ivashkiv, 2020).

In addition, there is important cross‐talk and cross‐regulation between nuclear factor kappa‐light‐chain‐enhancer of activated B cell (NF‐κB), a major transcription factor integrally involved in immune responses, with HIFs: HIF‐1α is an activator of NF‐κB and NF‐κB an activator of HIF‐1α (Rius et al., 2008). Reduction of HIF‐1α, however, may also reduce NF‐κB. HIF‐1α thus may limit excessive inflammation (Bandarra et al., 2015).

In summary, molecular pathways following inflammation and hypoxia are intricately linked and inter‐dependent. They can result in beneficial or detrimental consequences for mitochondria, depending on the hypoxic dose. Mitochondrial function in turn is integral in the host immune responses to SARS‐CoV‐2 infection and determines (and is determined by) further oxidative stress and inflammatory responses.

## 6 MAY ALTITUDE/HYPOXIC CONDITIONING BE BENEFICIAL DURING THE COVID‐19 PANDEMIC?

The cells primarily affected in COVID‐19 are epithelial cells, alveolar epithelial cells, vascular endothelial cells, and macrophages in the lung (Gordon et al., 2020). SARS‐CoV‐2 infection of these cells may cause pyroptotic cell death, which is characterized by the release of pro‐inflammatory factors and regional inflammation (Tay et al., 2020), mitochondrial dysfunction, and oxidative stress (Piantadosi & Suliman, 2017). Mitochondria of lung cells are required for efficient alveolar gas exchange and maintaining efficient ventilation (Cloonan & Choi, 2016; Piantadosi & Suliman, 2017). Mitochondrial damage thus may result in hypoxemia and in the worst case can cause direct respiratory failure. Associated oxidative stress will lead to the oxidation of molecules, including phospholipids, which have been detected in SARS‐patients (Imai et al., 2008) and may further aggravate inflammation. It is thus expected that all these effects, including oxidative stress, inflammation, and hypoxemia provoke HIF‐mediated remodeling of lung mitochondria, including the discussed glycolytic shift and pathological angiogenesis (Ackermann et al., 2020).

Together, these processes may also compromise coagulation regulation, which is important due to the correlation of coagulation abnormalities with COVID‐19 severity (Merad & Martin, 2020). Oxidative stress and oxidized phospholipids are furthermore involved in the upregulation of vascular glycolysis and together these conditions not only promote inflammation but are expected to be favorable for viral replication (Schnitzler et al., 2020).

Based on these observations, hypoxic conditioning may be a promising approach to reduce the risk and severity of SARS‐CoV‐2 infection in healthy individuals. In particular, increased endogenous antioxidant capacities, mitochondrial and immune system functions, as well as improvements of oxygen delivery systems (e.g., mediated via HIF‐induced EPO and VEGF) all suggest potential benefits for subsequent SARS‐CoV‐2 infection. However, the practicality of any preconditioning strategy is questionable and requires further investigation. In case of acute infection, exposure to hypoxia may be detrimental (see Figure 1) given the already sensitized systems on molecular and organ levels, in particular in states of high oxidative stress, inflammation, and mitochondrial dysfunction in lung and vasculature. As such, a careful COVID‐19 screening of athletes or patients is paramount before recommending altitude/hypoxic exposure (Carmody et al., 2020).

Intermittent conditioning is also known for being effective for COVID‐related comorbidities factors such as obesity (Sattar et al., 2020; Tartof et al., 2020) or low cardiorespiratory fitness (Zbinden‐Foncea et al., 2020) and may, therefore, be applied as a primary or secondary prevention measure.

**Figure 2:**
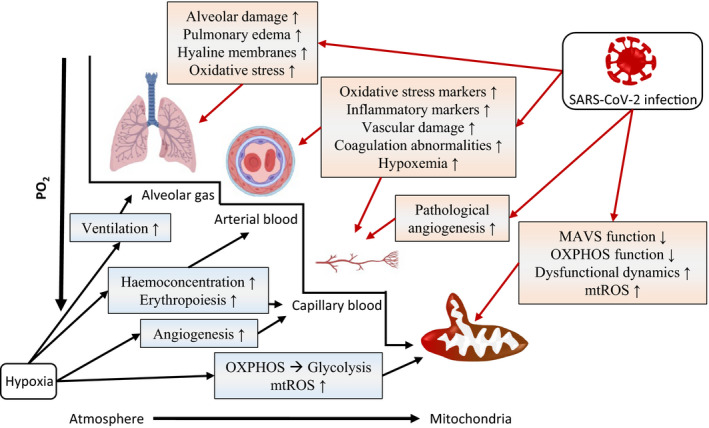
Potential effects of SARS‐CoV‐2 infection or a mild hypoxic stimulus on components of the oxygen cascade. Oxygen partial pressure (PO_2_) decreases along the oxygen cascade from inspired ambient air down to mitochondria, where oxygen serves as the final electron acceptor. A mild hypoxic stimulus induces adaptations to deal with reduced oxygen availability. Severe Acute Respiratory Syndrome Coronavirus 2 (SARS‐CoV‐2) infection may cause pathological alterations in response to damage to mitochondria, the respiratory, and the circulatory systems as a consequence of direct viral damage and the immune defense. The compromised oxygen supply system results in adaptations that partly overlap with adaptations to a mild hypoxic stimulus. MAVS – mitochondrial anti‐viral signaling complex, OXPHOS – oxidative phosphorylation, mtROS – mitochondrial reactive oxygen species

As shown in Figure 2, SARS‐CoV‐2 infection may exert adverse effects on oxygen uptake, supply systems, and extraction, resulting in mitochondrial dysfunction, oxidative stress, and compromised immune function with severe systemic consequences. Intermittent hypoxia may elicit adaptations with the potential to oppose SARS‐CoV‐2 infection‐related effects, via the capacity of hypoxic conditioning to optimize cellular oxygen supply, antioxidant systems, and mitochondrial function.

To summarize, intermittent hypoxic interventions have been shown effective in many clinical populations (Millet et al., 2016b) and can be prescribed as an anti‐hypertensive method (Serebrovskaya et al., 2008) or as an effective method for improving cardiorespiratory fitness (Fernandez Menendez et al., 2018) or the fat mass reduction (Kayser & Verges, 2013) in individuals with obesity. This approach may also be useful post‐infection since hypertension and obesity are identified as risk factors for COVID‐19 severity (Leiva Sisnieguez et al., 2020; Sattar et al., 2020; Tartof et al., 2020). More specific to respiratory diseases, there is accumulating evidence that intermittent hypoxia (as presented in section 4) may be effective as a means of hypoxic conditioning and possibly as a treatment in COPD, improving the respiratory control (Haider et al., 2009), exercise tolerance or decreasing the adrenergic responsiveness in these patients (Burtscher et al., 2009, 2010). However, we are aware that all possible treatments and prevention of disease discussed in the present review (e.g., intermittent hypoxia and hyperbaric oxygen), may be impractical and very costly to any health care system. There is an immense need to better understand their downstream signaling and achieve their benefits by other means, for example, pharmacologically.

## 7 CONCLUSION

The risks/benefits balance of altitude/hypoxic exposure and/or training for COVID‐19 patients remains difficult to appreciate but our current understanding of the five points discussed in the present review can be summarized as follows:

1. COVID‐19 pathogenesis and altitude adaptations may partly overlap on molecular and systemic levels in some aspects but there are crucial differences. COVID‐19 cannot be viewed as generally similar to high‐altitude illnesses,
2. Therefore, the optimal treatments are clearly different. This does, however, not exclude that some medications, for example, dexamethasone, and/or approaches, for example, HBOT, for high‐altitude illnesses hold promise for application in distinct aspects of COVID‐19,
3. It presently seems unlikely that altitude residence provides a protective effect against SARS‐CoV‐2 infection,
4. Several potentially interesting altitude‐related mechanisms (e.g., EPO, HIF‐1 pathway, mitochondrial enhancement possibly strengthening mitochondrial antiviral signaling) have been identified,
5. Intermittent hypoxia appears valuable to boost mitochondrial, anti‐oxidative, and immune capacities but with regard to COVID‐19 requires even tighter control of the patients’ responses to confer optimal benefits. Since hypoxic conditioning may be effective for COVID‐related comorbidities factors such as obesity or low cardiorespiratory fitness, it may be applied as a primary or secondary prevention measure, especially for persons at risk. Intermittent hypoxia may have deleterious effects, if temporally overlapping with SARS‐CoV‐2 infection.

To summarize, it seems unlikely that individuals going to high‐altitude areas would benefit from reduced risk and severity of SARS‐CoV‐2 infection. Conversely, high altitude sojourn at this time seems not to be associated with additional risks with respect to the pandemic—provided traveling is possible and safe and public health precautions are practiced. Hypoxic preconditioning performed for example as an altitude pre‐acclimatization strategy appears to be a promising way to improve immune responses, decrease inflammation, and better tolerate a “silent hypoxemia” state.

Finally, due to the novelty of the pandemic and despite the fast expending knowledge about it, at this stage, there are many unknowns with this virus from both a pathophysiology and epidemiology perspective. We have, therefore, to remain cautious regarding the effectiveness and practicality of any therapeutics recommended or dismissed until more research is conducted. Due to the ever‐changing state of understanding of COVID‐19, all points discussed in this review may be out of date at the time of its publication.

## CONFLICT OF INTERESTS

The authors declare no conflicts of interest related to the topic of this article.

## AUTHOR CONTRIBUTION

All authors contributed to the preparation and writing of the article. All authors read and approved the final version of the manuscript.

## AUTHOR CONTRIBUTIONS

Conceptualization, G.P.M. and J.B.; Methodology, G.P.M., M.B., and J.B.; Writing – original draft, G.P.M., M.B., and J.B.; Writing – Review and editing G.P.M., T.D., F.B., M.B., and J.B.
